# Investigating the use of machine learning algorithms to support risk-based animal welfare inspections of cattle and pig farms

**DOI:** 10.3389/fvets.2024.1401007

**Published:** 2024-08-13

**Authors:** Beat Thomann, Thibault Kuntzer, Gertraud Schüpbach-Regula, Stefan Rieder

**Affiliations:** ^1^Veterinary Public Health Institute, Vetsuisse Faculty, University of Bern, Bern, Switzerland; ^2^Identitas AG, R&D, Bern, Switzerland

**Keywords:** data-driven, random forest, animal health, animal welfare, monitoring

## Abstract

In livestock production, animal-related data are often registered in specialised databases and are usually not interconnected, except for a common identifier. Analysis of combined datasets and the possible inclusion of third-party information can provide a more complete picture or reveal complex relationships. The aim of this study was to develop a risk index to predict farms with an increased likelihood for animal welfare violations, defined as non-compliance during on-farm welfare inspections. A data-driven approach was chosen for this purpose, focusing on the combination of existing Swiss government databases and registers. Individual animal-level data were aggregated at the herd level. Since data collection and availability were best for cattle and pigs, the focus was on these two livestock species. We present machine learning models that can be used as a tool to plan and optimise risk-based on-farm welfare inspections by proposing a consolidated list of priority holdings to be visited. The results of previous on-farm welfare inspections were used to calibrate a binary welfare index, which is the prediction goal. The risk index is based on proxy information, such as the participation in animal welfare programmes with structured housing and outdoor access, herd type and size, or animal movement data. Since transparency of the model is critical both for public acceptance of such a data-driven index and farm control planning, the Random Forest model, for which the decision process can be illustrated, was investigated in depth. Using historical inspection data with an overall low prevalence of violations of approximately 4% for both species, the developed index was able to predict violations with a sensitivity of 81.2 and 79.5% for cattle and pig farms, respectively. The study has shown that combining multiple and heterogeneous data sources improves the quality of the models. Furthermore, privacy-preserving methods are applied to a research environment to explore the available data before restricting the feature space to the most relevant. This study demonstrates that data-driven monitoring of livestock populations is already possible with the existing datasets and the models developed can be a useful tool to plan and conduct risk-based animal welfare inspection.

## 1 Introduction

Animal health and welfare is a subject of ever-growing importance. In this context, the Smart Animal Health (SAH) project was initiated in Switzerland, aiming to develop data-driven methods for assessing animal health and welfare for different livestock species (1). The literature review on data- and animal-based indicators revealed large differences between animal species, concerning data availability and reliability of indicators (2–7). Although high correlations with animal health and welfare have been obtained for some indicators, it was concluded that a combination of data-based indicators and on-farm assessments is currently required for a comprehensive estimation of the health and welfare status at the farm level (1). The review on available precision livestock farming (PLF) technologies, which can provide a time-saving and objective alternative to the manual collection of indicators, also identified substantial differences between animal categories and large discrepancy between scientifically validated and commercially available PLF systems (8–10). In contrast to the “classical” and established animal-based indicators described in the reviews above, which are then compiled into protocols for a comprehensive assessment of the health status, the part of the project described in the study focused on the integration of multiple data sources to discover and study previously unrecognised correlations and indicators that can be used as proxies.

Animal data are stored in various databases that reflect the complex livestock landscape. Studying these databases and deriving actionable information can contribute to improved animal health and welfare (11, 12). Data-driven analyses may also support risk-based surveillance (13, 14). For decades, the collection of data in the livestock sector in Switzerland and Liechtenstein has been constantly expanded (in the following, the term Switzerland should be construed including the Principality of Liechtenstein due to the common agricultural policies in the two countries). The collection of data covers multiple aspects of the general agriculture policy and public veterinary issues (15). Data are used to guarantee the traceability (16), safety, and quality of animal products (17), monitor and control animal health and welfare at individual and population levels (18), and allocate direct subsidies to livestock farmers (19).

The analysis of animal databases reveals correlations between proxy values and indicators (20) or paves the way for the development of farm digital twins (i.e., a digital simulation of the farm), which would continuously monitor or predict metrics (21, 22). On the other hand, common methods for animal welfare evaluation are based on data directly recorded on the farm, such as the Welfare Quality® protocol (23). However, there is a trend towards an increased use of data that is routinely and widely captured, or/and, to use so-called iceberg indicators that reduce the number of parameters and resources needed (24–26). Other data-driven methods have successfully predicted disease risk in dairy cattle and revealed previously unknown relationships between proxy data and animal health and/or welfare (27–29). Currently, descriptive statistics that show the evolution of the animal populations are publicly available (30), and there is much more descriptive statistics to be extracted. Third-party non-animal-centric datasets can be added to give more insights, such as geography (29), transport durations, or climate data.

In this study, we mainly focused on livestock-specific data. Based on proxy data, we built a proof-of-concept decision-making tool to assess animal welfare in relation to the Swiss legislation at the farm-level for cattle and pig holdings. The risk index modelled the results of the regularly conducted on-farm animal welfare inspections using supervised classifiers by interconnecting and exploiting multiple databases. In contrast to unsupervised learning, in supervised learning, an algorithm must be trained with labelled data which are divided into classes, and the unlabelled data are then assigned to these existing classes. The risk index does not aim at replacing the on-site controls but merely proposes a list of farms to monitor and visit in priority.

## 2 Materials and methods

**Figure 1:**
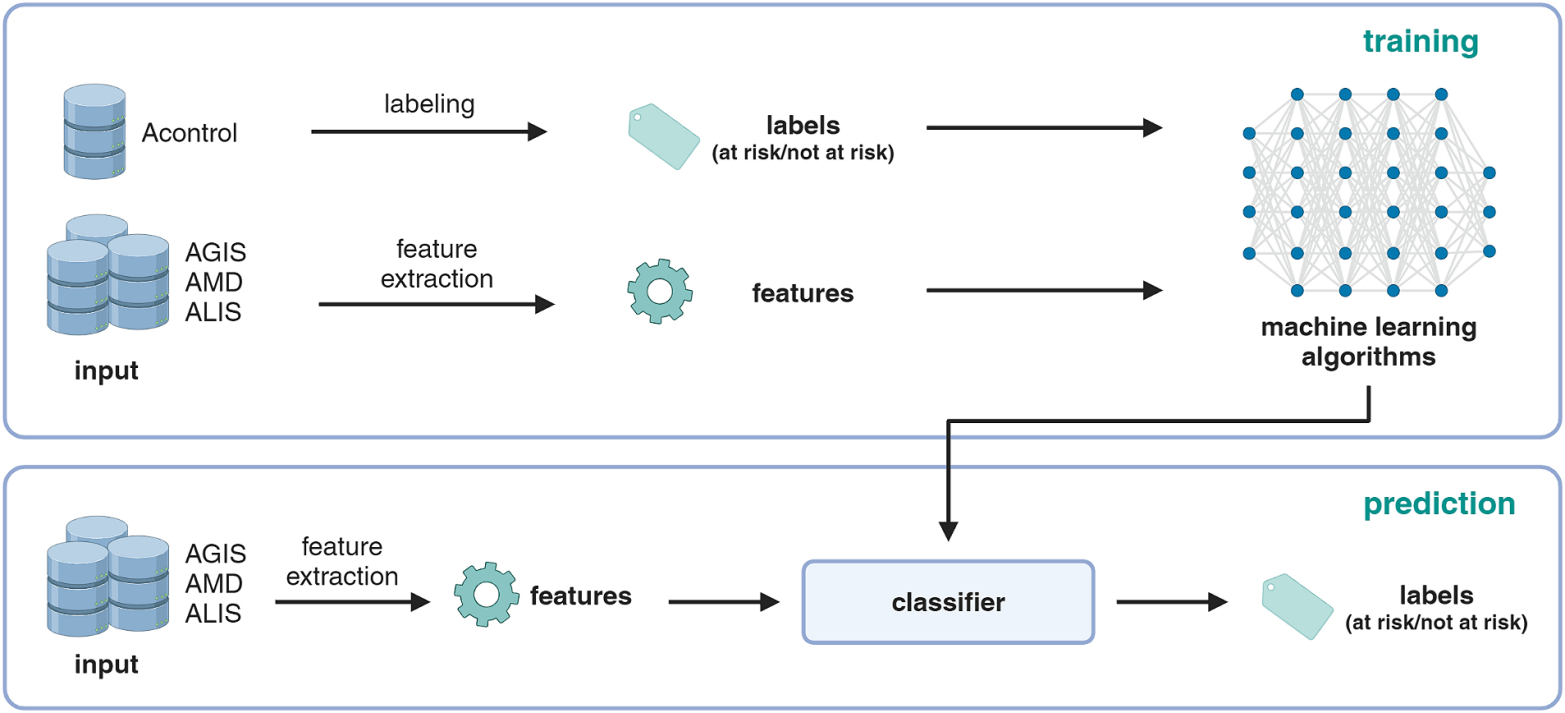
Illustration of the information and data flow and the development steps of the risk index. Created with BioRender.com.

The development process of the risk index and the data sources involved are shown in Figure 1. Data from four individual databases were interconnected: Acontrol, AGIS, AMD, and ALIS. Data from previous on-farm welfare inspections in Acontrol were used as labels to calibrate a binary welfare index (at risk/not at risk). The features used for prediction (i.e., proxy variables) were extracted from the three other databases AGIS, AMD, and ALIS. Four different machine learning algorithms were applied and compared. In the following, the individual steps and databases are explained in detail: (i) data sources, (ii) labels and features, and (iii) classification algorithms.

### 2.1 Data sources

Livestock data are stored in various databases that are usually not interconnected but share common unique identifiers for animals and farms. Some of the records are stored in public databases, where content and access are regulated under public law, and some records are stored in private infrastructures (31). These databases might register information with different granularity. We did not restrict previous data sources based on their aims or usage. However, we selected only data sources, where data are routinely collected from all livestock farms, to achieve sufficient quality, coverage (i.e., the fraction of reporting farms for a given parameter), and availability (i.e., accessibility). We granted a pseudonymised access to the databases described below. However, for data privacy and ownership reasons, we did not have access to all parameters. In the following, the data sources used are briefly described.

#### 2.1.1 Control data system (Acontrol)

The Acontrol database contains the results of the mandatory on-farm animal welfare inspections among other standardised controls in the field of primary production (e.g., milk hygiene and documentation of the use and storage of veterinary medicines). These data served as the basis of the labelling procedure (see Section 2.2.1), i.e., this is the status to be predicted by the classification algorithm. Each farm has to be visited and inspected at least every 4 years (31). It is recorded whether a farm had any violations at the various control points. The individual control points are grouped into categories. An overview of these control categories is shown in Table 1. For cattle farms, the on-farm welfare inspection consists of 18 control categories, such as minimal dimensions and housing requirements, light and air quality, water supply, and injuries and animal care, whereby the latter control category includes sub-control points for lesions, lameness, body condition, or dirty animals. The detailed inspection protocol can be obtained from the Federal Food Safety and Veterinary Office (FSVO) (32). For pigs, the welfare inspection consists of 15 control categories, which is similar to those in cattle (33). Additional information and guidelines on the on-farm inspections are available in the FSVO (34). The welfare status was thus defined as the compliance/violation of control points in accordance with the corresponding regulations. The Acontrol database is administered by the FSVO and the Federal Office for Agriculture (FOAG).

**Table 1:** Overview of the on-farm animal welfare inspection control categories for cattle and pig farms.

Cattle	Pig
1. Education and training 2. Minimum dimensions 3. Occupancy of the stables 4. Flooring 5. Lying area 6. Control devices to influence animal behaviour 7. Lighting 8. Air quality, fresh air supply and noise 9. Water supply 10. Feeding area in loose housing barns 11. Calving pen in loose housing barns 12. Calf housing: individual keeping, visual contact and feeding 13. Tethering in tie-stall barns 14. Movement in tie-stall barns 15. Permanent outdoor keeping 16. Injuries, animal care including hoof care 17. Interventions on the animal 18. Miscellaneous	1. Education and training 2. Minimum dimensions 3. Occupancy of the stables 4. Flooring and lying area 5. Control devices to influence animal behaviour 6. Lighting 7. Air quality, fresh air supply and noise 8. Temperature 9. Water supply 10. Activity, bedding and nesting material 11. Individual keeping 12. Permanent outdoor keeping 13. Injuries, animal care including hoof care 14. Interventions on the animal 15. Miscellaneous

Based on proxy variables from other data sources, the risk index predicts farms with an increased likelihood of violating the requirement in these categories.

#### 2.1.2 Agricultural policy information system (AGIS)

The AGIS database (DE: *Agrarpolitisches Informationssystem*) by FOAG is a central tool to administer the public direct subsidies to farmers in Switzerland and has a high coverage and data quality. It also serves as a hub for coordinated and harmonised use of administrative data on farms, primarily at the federal level (31). The system centralises farm records, such as structural data (e.g., farm type and production system, surface and land use, type of livestock species kept, or number of heads by species and category) and direct subsidy programmes. These programmes include two animal welfare/ethological programmes that are funded by the federal government: “particularly animal-friendly housing systems” (BTS; DE: *Besonders tierfreundliche Stallhaltungssysteme*) and “regular outdoor access” (RAUS; DE: *Regelmässiger Auslauf im Freien*). The BTS and RAUS programmes proved to be of particular importance in this study. The BTS/RAUS ethological programmes specify higher standards regarding animal welfare compared with those set out in the basic animal welfare legislation. The BTS programme requires animals to be kept in larger and more structured housings, while the RAUS programme demands that animals have access to outdoor areas more often (35). Moreover, the granting of direct payments for farmers per default requires that the beneficiary fulfils the requirements of the ecological performance certificate (OLN; DE: *Ökologischer Leistungsnachweis*) on the entire farm (36). The OLN requirements are aimed, for example, at increasing biodiversity, limiting air pollution, and balancing nutrient and fertiliser flows, crop rotation, and soil protection. Participation in optional OLN programmes is also recorded in AGIS.

#### 2.1.3 Animal movement database (AMD)

The AMD (DE: *Tierverkehrsdatenbank TVD*) centralises data on the following species: bovine, goat, sheep, swine, equid, poultry, camelids, and game animals. It has a legal mandate to provide traceability data for individual animals (in the case of ruminants and equids) or groups of animals (pigs and poultry). For all other species, only the livestock unit is known. For those species with individual animal recordings, the total of stays and keeper/owner information can be extracted, starting from the birth or import notification to the final culling or export notification. The AMD is developed, maintained, and operated by Identitas AG under the guidance of FOAG and FSVO. Not all species are covered with the same level of details or accuracy. Available data include animal’s identity, its parents, a series of phenotypes, and major events (e.g., birth, departure from and arriving at a holding, and culling). Poultry, small ruminants, and equids were not considered in this study. The presence of these animals on a farm was used as an input parameter. Group notifications describe the nature of the event (arriving at a new farm or a processing facility) and the number of animals in the group. Further information is recorded against the AMD, such as disease status or information about the farm (e.g., geographical location). AMD has an excellent coverage. A comment on the overall data quality is difficult because it varies for every attribute. It ranges from very good for the basic data and common notifications to average for the precise geographic localisation of holding and poor for additional optional attributes, which also have poor coverage, such as reasons for animal culling.

#### 2.1.4 Laboratory information system (aRes, formerly ALIS)

This FSVO system centralises laboratory data from the approved laboratories of the public veterinary service. The data include diagnostic results on notifiable animal diseases according to the Swiss animal disease ordinance (37), including zoonoses that are carried out on behalf of the federal and cantonal veterinary services as part of disease surveillance and control programmes or animal movement regulations. For this study, data on mandatory abortion examinations in cattle (i.e., for infectious bovine rhinotracheitis, bovine viral diarrhoea, brucellosis, and coxiellosis) were included in the models (38).

#### 2.1.5 Further databases

In this study, two further databases were of great interest: the information system on antibiotics in veterinary medicine (IS-ABV) and the meat inspection database (FLEKO). However, both were introduced during the research project only, and therefore, could not be considered as sources for input parameters due to low coverage, restrictive data security, and, so far, insufficient data quality.

#### 2.1.6 Data extraction

The development of the risk index was limited to the farms of cattle and pigs for data coverage and quality reasons. Data were anonymised prior to processing according to data processing agreements with the competent authorities. A pseudonymisation procedure was agreed to meet the need for precise and unbiased data points. The identification codebook was built by the FSVO and was kept confidential. The FSVO also pseudonymised data from the public data sources of the federal offices and the data were anonymous for us. The time window of the data selected in this study is from January 2014 to the end of October 2019, spanning almost 6 years. During this time window, more than 48,700 holdings (including summer holdings and other temporary holdings) did actively register cattle. A total of 15,800 animal holdings were involved in the trade of pigs during the 6 years.

### 2.2 Labels and features

Data were homogenised, formatted, and normalised to be used as features (i.e., input parameters) or labels (i.e., the objective for each holding) for machine learning algorithms. Some of the data were categorical and others were numerical. Most were time series with varying time steps, which may exhibit strong and complex seasonal variations. In this section, we outline the main pre-processing techniques. We emphasise that our goal was to predict an animal welfare status at the farm level. The label “welfare status,” or risk index in the following, should reflect the findings of the on-farm inspections and be based on proxy data that were not collected during those visits.

#### 2.2.1 Label preparation

Based on historical results from on-farm inspections recorded in the Acontrol database, a scalar metric was built. According to the Swiss Animal Welfare Act and Ordinances, the welfare status was thus defined as the compliance/violations of control points (39–41). All control points were treated equally, and the various sub-points (e.g., lameness, body condition) were not labelled individually. Follow-up control visits were disregarded due to previous violations. A weighting scheme was applied to diminish the weight of announced inspections (factor of 0.8) compared with unannounced inspections (factor of 1). Moreover, if more than one inspection occurred during the selected time window, the importance of older inspections was also reduced by an exponential weighting scheme. Finally, the label for the risk index is a binary value depending on the fraction of inspections with registered violations over the total number of (relevant) controls. For a holding to be considered “at risk,” this proxy should be greater than or equal to 0.5.

#### 2.2.2 Feature extraction

There are a number of different formats for the input data. Features were chosen to be scalars and feature extraction was designed such that dimensionality was reduced; time series were aggregated or classified into data-driven categories. The participation of a holding in individual programmes, such as “grassland-based dairy production” or “biodiversity promotion areas” (42), was grouped by larger themes (e.g., other OLN). Categorical data (e.g., canton or the holding type) were encoded using a one-hot scheme (i.e., a categorical feature containing *K* possible values, which is transformed into *K* binary features). Distributions were encoded using simple descriptive statistics.

For cattle, herd descriptions per holding, age, and sex were prepared. This included head count, mean age, number of different breeds, fraction of breed type (dairy, beef, or dual), and fraction of non-technical names given to livestock. Notifications to the AMD registry were counted per holding and per type. Animal age at notification and notification delays between event and reporting were described using 5, 25, 50, 75, and 95 percentiles. Moreover, the fraction of heifers and cows was estimated for every notification type. AGIS provides yearly head count per animal category, which was included for analysis of both cattle and pig holdings. The evolution of the number of cattle per category depends on the holding type and practises. Aggregating this information without considering the dynamics of the herds would result in a loss of information. Using the Euclidean *k*-means clustering algorithm (43), these time series were transformed into categorical features.

For pigs, transport notifications were extracted from the AMD. As for cattle, we extracted notification type and delay description. Origin and destination of the transports were used to compute the distances and travel times (44). Measured transport duration will be routinely reported in the AMD. However, currently, only the origin and destination are notified.

Non-categorical features were normalised. The average or percentile values of the time series were normalised by the total of the corresponding categories, e.g., each cattle category was normalised by the total number of animals. In the databases that were available, some parameters were highly incomplete, with some records having less than 50% coverage. Missing data (e.g., many holdings lacked an entry for units of standard workforce) were replaced by a 0, which does not put the missing parameter in excessive focus during the analysis. There are a few different reasons for values to be missing, such as incorrect values entered manually; withholding data propagation from collection to the final database was not yet fully implemented. A missing value can also be informative in this context. Finally, all features underwent standard scaling, which is imposing a zero mean and unit variance.

An overview of feature themes and groups is presented in Tables 2 and 3. In addition, a more detailed description of the most important features is presented in the results section.

**Table 2:** Overview and breakdown of the 297 features for cattle holdings with features categorised by database and group.

Source database	Theme	Group	Feature number	RF feature importance
**AMD**			**227**	**66.5%**
	*Herds*		*63*	*21.4%*
		Monthly head count by category	15	8.5%
		Head count time evolution category	28	1.7%
		Mean age by category	14	7.2%
		Cattle breed diversity	5	3.3%
		Animal named	1	0.8%
	*Cattle movements*		*109*	*38.3%*
		Arrival notifications	8	4.9%
		Arrival notifications discipline	5	2.8%
		Departure notifications	13	5.6%
		Departure notifications discipline	5	3.3%
		Reason for departure	5	2.3%
		Births/stillbirth notifications	11	3.0%
		Births/stillbirth notifications discipline	11	2.6%
		Lost cattle	7	1.9%
		Death notifications	15	5.2%
		Death notifications discipline	10	2.6%
		Other notifications (on-farm slaughter, imports, exports, count)	14	1.1%
		Other notifications discipline	5	3.2%
	*Holdings*		*55*	*6.8%*
		Production type	14	2.2%
		Permission to hold other species	7	1.9%
		Geographical location (canton, agricultural region)	34	2.8%
**AGIS**			**69**	**33.2%**
	*Herds*		*22*	*7.6%*
		Yearly population (all species)	12	4.3%
		Population evolution (all species)	10	3.3%
	*Programmes*		*45*	*24.0%*
		BTS	12	2.9%
		RAUS	22	7.1%
		Others OLN	11	14.0%
	*Holdings*		*2*	*1.6%*
		Standard Workforce	1	1.0%
		Farm surface	1	0.6%
**ALIS**	Monitoring	Abortions	**1**	**0.3%**

Feature importance is based on the Random Forest (RF) classification. The bold numbers are the sum of the categories in italics (themes) in each source database. The numbers in italics show the sum of the numbers in normal font (groups) in each source database.

**Table 3:** Overview and breakdown of the 179 features for pig holdings with features categorised by database and group.

Source database	Theme	Group	Feature number	RF feature importance
**AMD**			**110**	**45.1%**
	*Pig movements*		*55*	*35.8%*
		Transport to slaughterhouses	3	3.6%
		Transport to slaughterhouses computed duration	5	6.1%
		Transport to slaughterhouses notification discipline	5	1.7%
		Transport to another holding	6	4.1%
		Transport to another holding computed duration	10	9.7%
		Transport to another holding discipline	10	8.6%
		Notifications evolution	16	2.0%
	*Holdings*		*55*	*9.3%*
		Production type	14	2.4%
		Permission to hold other species	7	3.7%
		Geographical location (canton, agricultural region)	34	3.2%
**AGIS**			**69**	**54.9%**
	*Herds*		*22*	*15.4%*
		Yearly population (all species)	12	10.2%
		Population evolution (all species)	10	5.2%
	*Programmes*		*45*	*37.0%*
		BTS	12	3.1%
		RAUS	22	7.0%
		Others OLN	11	26.9%
	*Holdings*		*2*	*2.5%*
		Standard workforce	1	1.5%
		Farm surface	1	1.0%

Feature importance is based on the Random Forest (RF) classification. The bold numbers are the sum of the categories in italics (themes) in each source database. The numbers in italics show the sum of the numbers in normal font (groups) in each source database.

#### 2.2.3 Description of the consolidated dataset

The consolidated datasets containing the different features were used as input data for the applied classification algorithms. For cattle, out of the 36,904 holdings with at least one record in Acontrol, 1,437 (prevalence of 3.89%) showed ≥50% of violations and were therefore classified at risk. Each holding was described by 297 features. For pigs, out of the 2,432 holdings with at least one record in Acontrol, 110 (prevalence of 4.52%) showed ≥50% of violations and were classified at risk. For pig holdings, there were 179 features. The datasets were, thus, unbalanced. All algorithms were trained on balanced training sets. To include more variability in the training set, we chose to oversample the category at risk by a factor of six.

### 2.3 Classification algorithms

The risk index as described above is a Boolean quantity, and consequently, the computation of the index is therefore a binary-class classification task. The scikit-learn Python library (45) implementation was used for all, but the artificial neural networks (ANNs) for Keras (46) was used.

Random Forest (RF) was used as baseline because of the low number of meta-parameters and interpretability. We built a forest of 500 decision trees using the entropy criterion. ANNs with the following architecture used two 30-neuron hidden layers with rectified linear unit activation and a classification layer with a sigmoid activation function yielding a scalar. The network was trained with binary cross-entropy loss and a L_1_ regularisation term using the Adam optimiser. The choice of this method was justified by good performance and flexibility. However, ANNs are notoriously difficult to inspect. Other algorithms such as support vector machines (SVM, or SVC, with a radial basis function kernel) and logistic regressions were tested. Finally, a compromise classifier was built which considers the answers of all models and weights the score according to the performance of each individual algorithm. This procedure is referred to as a committee voting system or committee in short.

For training, validation, and testing of the algorithms, three independent sets were used, which were divided into training (70%), validation (10%), and test (20%). Between each classification experiment, all sets were randomly drawn to ensure that classifiers were not trained on the same subsets. All of the methods mentioned above (except ANN) have some built-in decision thresholds, which are usually set at 0.5 of the classification score. Different heuristics can be selected to optimise the decision threshold. We experimented with three different methods: (i) maximising *F*_1_-score, (ii) imposing a false positive rate (FPR) of 20%, and (iii) maximising the value “*F*_1_-score—0.1FPR.” We selected the compromise (option iii) to maintain the FPR at a relatively low level while aiming for the best performances.

Due to the stochastic nature of the training and the relatively low number of training samples, the classification was repeated 1,000 times. This resolved the training variations in the final performances of the classifier and allowed for the standard deviation of the training to be evaluated.

## 3 Results

### 3.1 Risk index for cattle holdings

**Figure 2:**
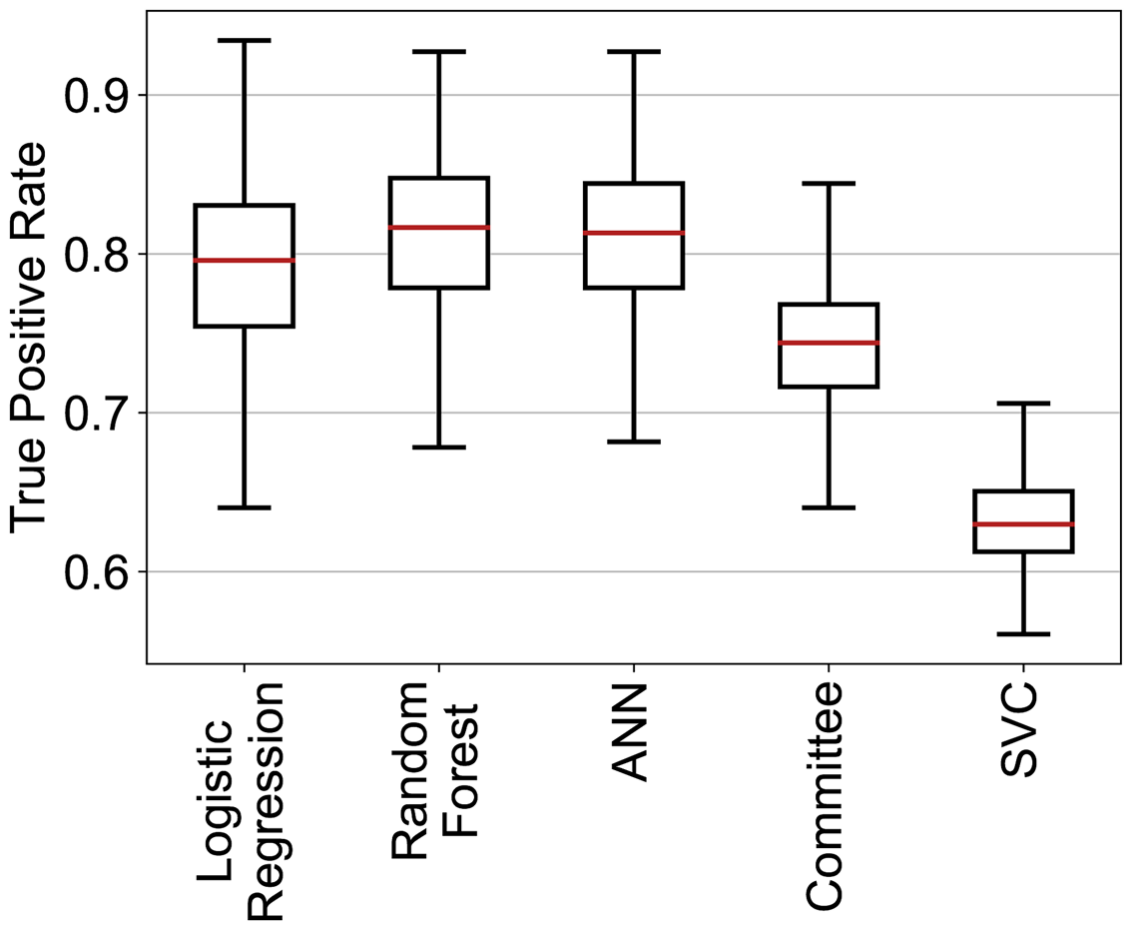
True positive rates of the different classifiers for the cattle holding risk index.

**Figure 3:**
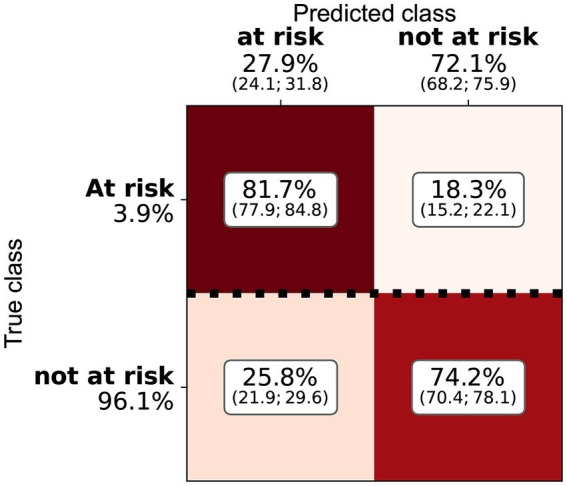
Confusion matrix for the random forest and the cattle holding risk index, indicating the median, Q1, and Q3 relative importance of the different classes. The elements are normalised by the number of true class examples (marked by the heavy dotted line).

Three of the algorithms applied showed similar performance (ANN, RF, and logistic regression—see Table 4 and Figure 2). Several architectures for ANNs have been explored, most exhibiting the very similar performance. RF and ANN tended to show a slightly better performance because of the smaller variation of their metrics. SVM showed a significantly higher precision but a much lower sensitivity. SVM could be considered a more conservative method. As the sensitivity was substantially lower, many at-risk holdings were not correctly classified. The confusion matrix for RF (Figure 3) shows that the median sensitivity was 81.7%. Individual trainings reached between 78 and 85% at the 25 and 75 percentile levels. RF reached a mean precision of 11.5% and accuracy of 74.1%.

**Table 4:** Mean and standard deviation of the performance metrics for cattle holdings.

	Logistic regression		SVC		ANN		RF		Committee	
Metrics	Mean	Std	Mean	Std	Mean	Std	Mean	Std	Mean	Std
Accuracy	0.729	±0.062	**0.858**	±**0.006**	0.745	±0.048	0.741	±0.053	0.818	±0.022
Sensitivity	0.790	±0.057	0.630	±0.029	0.811	±0.047	**0.812**	**±0.054**	0.742	±0.041
Precision	0.108	±0.002	**0.162**	**±0.007**	0.115	±0.002	0.115	±0.017	**0.162**	**±0.012**
*F*_1_ score	0.189	±0.025	**0.257**	**±0.011**	0.201	±0.022	0.201	±0.024	0.241	±0.015
AUC	0.833	±0.011	0.749	±0.014	0.844	±0.012	**0.854**	**±0.010**	0.836	±0.011

The best value in each line is highlighted in bold.

SVC, Support Vector Machine; ANN, Artificial Neural Network; RF, Random Forest; AUC, Area under the curve; std, standard deviation.

**Figure 4:**
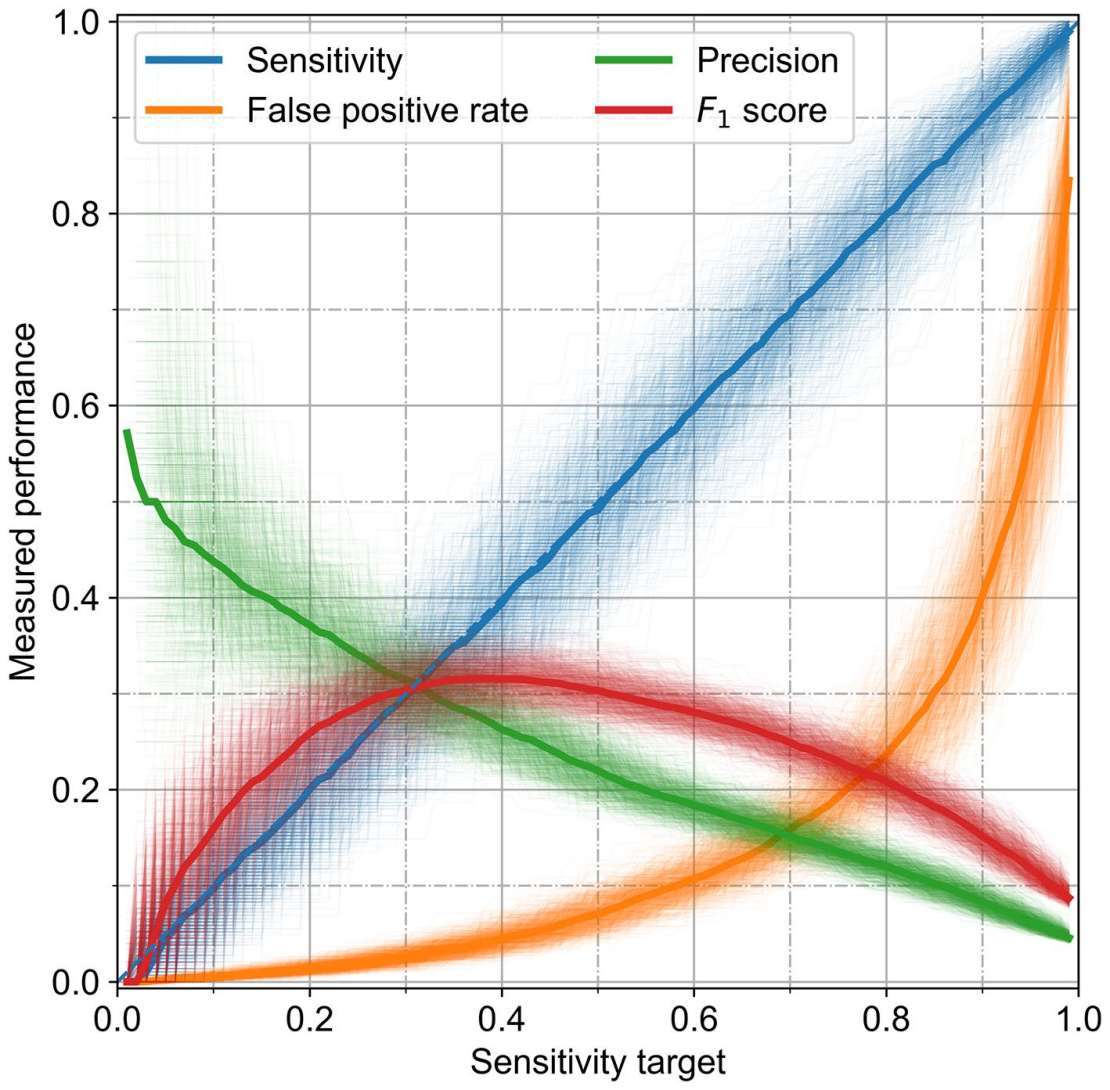
Performance of the random forest for the cattle holding risk index. The performance metrics are presented as a function of the classifying heuristic, which aims to achieve a given sensitivity. Solid thick lines are the median of the thin, partially transparent lines representing all runs.

We chose to set a compromise heuristic between precision and sensitivity. This heuristic reached the best possible performance for all classifiers. Figure 4 shows the different performance metrics as a function of target sensitivity for the RF. The algorithms could reach the performance set. At its maximum, the *F*_1_ score can reach values up to 32%, with a precision of 25%, but a low sensitivity of 40%. Individual runs showed a good similarity with the median performance; the standard deviations are presented in Table 4. RF performed better for any sensitivity chosen than the other algorithms, as it has a higher precision and lower false positive rate.

### 3.2 Risk index for pig holdings

**Figure 5:**
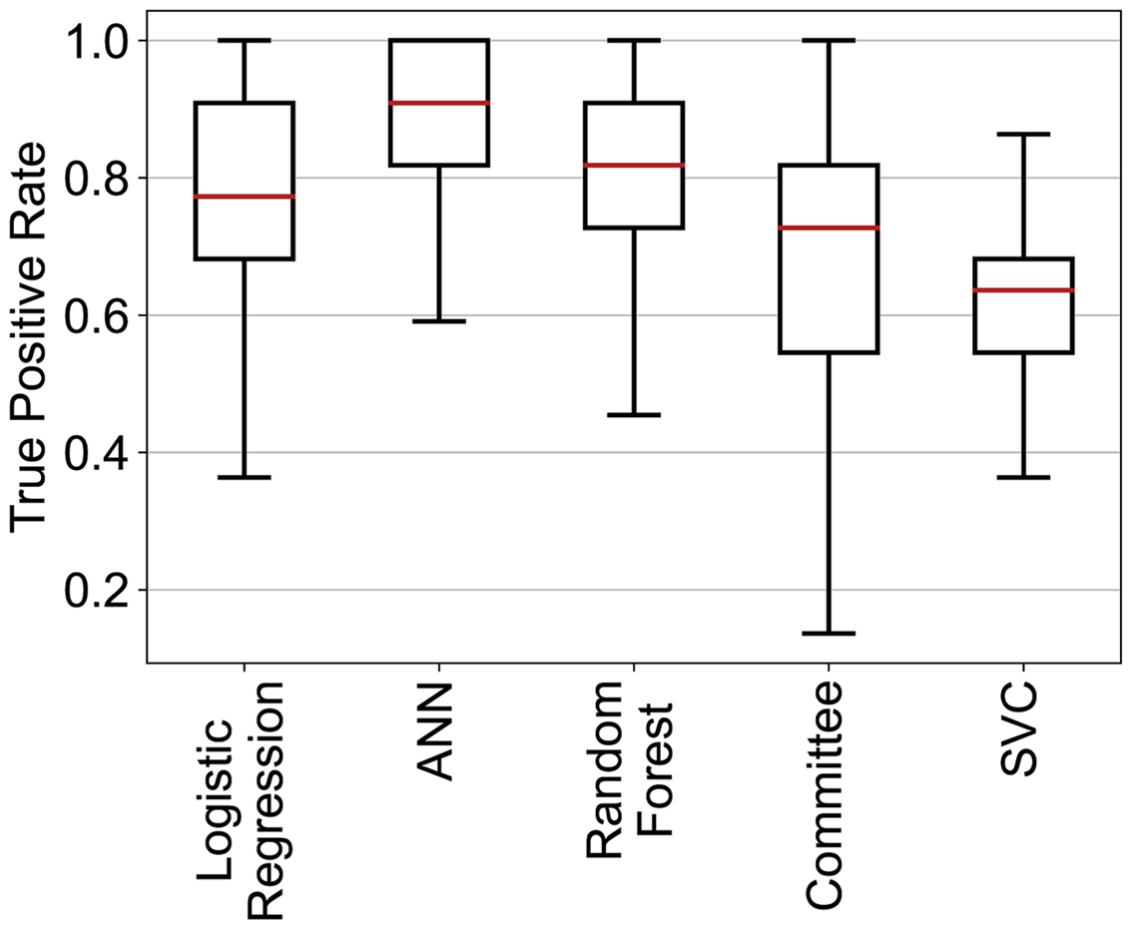
True positive rates of the different classifiers for the pig holding risk index.

**Figure 6:**
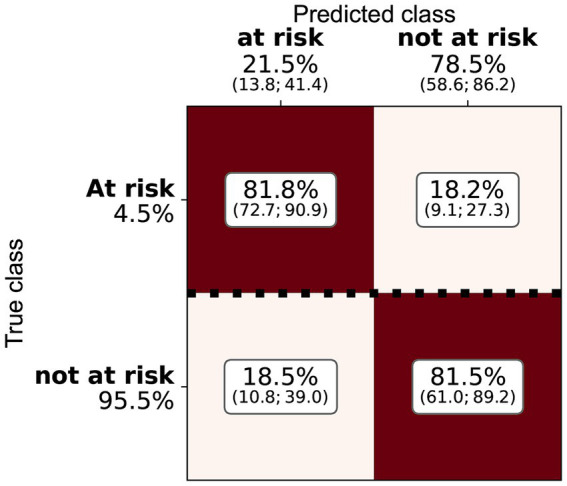
Confusion matrix for the random forest and the pig holding risk index, indicating the median, Q1, and Q3 relative importance of the different classes. The elements are normalised by the number of true class examples (marked by the heavy dotted line).

The variability of the performance metrics was higher for pigs than that in cattle (Figure 5). As shown in Table 5, SVC had a very high mean accuracy score of 90.6%, but the sensitivity of 62.0% was much lower than the other methods. RF revealed, as for cattle, the best trade-off between sensitivity (79.5%) and precision (18.0%, Figure 6). The accuracy of RF reached 73.1%. Thus, the results for cattle and pigs were very similar. The main performance difference with the classifiers for cattle holdings was the run-to-run variability of the methods.

**Table 5:** Mean and standard deviation of the performance metrics for pig holdings.

	Logistic regression		SVC		ANN		RF		Committee	
Metrics	Mean	Std	Mean	Std	Mean	Std	Mean	Std	Mean	Std
Accuracy	0.665	±0.184	**0.906**	**±0.014**	0.564	±0.367	0.731	±0.206	0.846	±0.133
Sensitivity	0.770	±0.153	0.620	±0.102	**0.839**	**±0.250**	0.795	±0.160	0.632	±0.285
Precision	0.120	±0.053	**0.270**	**±0.045**	0.139	±0.088	0.180	±0.097	0.230	±0.095
*F*_1_ score	0.200	±0.066	**0.374**	**±0.056**	0.210	±0.130	0.271	±0.106	0.285	±0.137
AUC	0.821	±0.043	0.770	±0.050	0.860	±0.054	**0.866**	**±0.053**	0.858	±0.047

The best value in each line is highlighted in bold.

SVC, Support Vector Machine; ANN, Artificial Neural Network; RF, Random Forest; AUC, Area under the curve; std, standard deviation.

### 3.3 Feature importance

#### 3.3.1 Cattle

**Figure 7:**
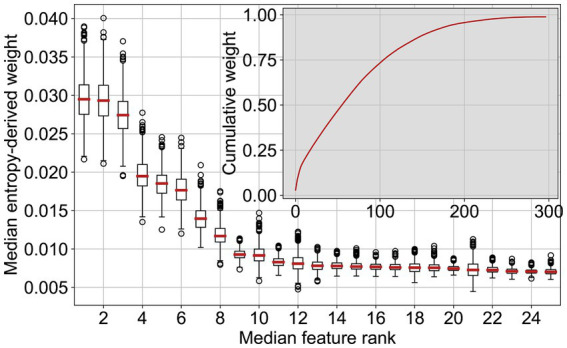
The 25 most important features for the random forest cattle classifier, sorted by their median importance weight and the cumulative feature weight.

Listing the most important RF features for cattle revealed that the top 50 features account for almost 50% of the decision weight (Figure 7). The box plots in Figure 7 represent the run-to-run variability of the feature importance for the 25 most important features. While there was some variation, the order of the features, or the order of their groups aggregated by theme, did not significantly change. The feature importance for all groups of features for cattle is presented in Table 2. Participation in production system programmes (e.g., BTS, RAUS, and OLN) considerably correlated with a good health and welfare status, i.e., the compliance of control points in on-farm welfare inspections. The features associated with these programmes (45 features) accounted for 24% of the decision. In the following, features are summarised in groups and values are mentioned in parenthesis representing group importance. The order in which the features are cited follows individual feature importance in the decision tree. After production system programmes, some structural features were next in order of importance. The standard workforce was one of the most important features (1% of the decision, one feature) despite being incomplete. Whether the farm was registered as an all-year husbandry (DE: *Ganzjahresbetrieb*) was also an important binary flag (1%, one feature). The first feature directly connected to animals, concerning the head count and time evolution per category (10.2%, 43 features), age per category (7.2%, 14 features), departure notifications and the age at departure (5.6%, 13 features), the discipline of departure notifications (3.3%, 5 features), and their reasons (2.3%, five features). Some of these important features also pertain to the breed diversity in the herd (3.3%, five features). The proportion of animals with non-technical names also ranked high (0.8%, one feature). The fraction of animals that die is the first feature, which is directly related to health and welfare (5.2%, 15 features), followed by the fraction of lost cattle, i.e., cattle that departed a holding (1.9%, seven features) but, according to the records in the AMD, never arrived at another holding or abattoir. Features related to stillbirths ranked relatively low and might be slightly less meaningful than lost animals (3.0%, 11 features). The surface of the holding (DE: *landwirtschaftliche Nutzfläche*) was important (0.6%, one feature).

#### 3.3.2 Pigs

An overview of the feature importance for all groups of features for pigs is presented in Table 3. The feature analysis for pigs was similar to cattle: a few important features and a long tail of much less significant features. The most important features are grouped into what can be called animal welfare and ecological programmes (37%, 45 features), which comprise three groups, namely, BTS, RAUS, and “other OLN.” Next, the standard workforce (1.5%, one feature) and farm surface (1%, one feature) played key roles. The animal species other than pigs (8.4%, 10 features) that are held on the holding were among the most important features, in particular the presence of cattle. Furthermore, the transport notifications, particularly the fraction of transports to slaughterhouses (3.6%, three features), were relevant. The same is true for the computed transport duration to either slaughterhouses or other holdings (15.8%, 15 features). The flux of pigs arriving at or departing from a holding was also important (4.1%, six features). For all of the transport notifications, the transport durations were more relevant than the notification discipline (10.3%, 15 features).

### 3.4 Sensitivity analysis

To test the sensitivity of the performances to the number of features, we selected only the 100 most important ones in the cattle model according to the list in the above paragraph, which account for more the 75% of the decision, and re-run the classification. The resulting performance metrics were statistically consistent with the all-feature runs. Moreover, to regress the true fraction of health and welfare violations (instead of classifying the holdings between at risk and not at risk), the experimental extension of the method did not show any improvement on the results presented in the previous sections. The additional analyses carried out for the comparison of feature importance of the logistic regression revealed that the feature order is roughly similar to RF for pigs. This was expected because of the very similar performances. For cattle experiments, we observed that the type of notifications, in particular the ones linked to mortality, and the production type were the most important features for logistic regression.

## 4 Discussion

Based on the analyses, RF was determined to be the most suitable algorithms, as the classifications can be explained and the performance loss for the individual metrics is minor compared with other algorithms tested. Moreover, RF has shown high sensitivities towards welfare violations, allowing the detection of a high number of true positive cases, which is important in the context of the wider objectives of this study. However, due to the overall low prevalence of the violations for both animal species (at roughly 4%), a small but non-zero false positive rate leads to multiple false positive outcomes. As a result, the overall precision is relatively low, ranging from 11 to 15% for cattle farms and 18–20% for pig farms (Tables 4 and 5). Nevertheless, the precision can be increased by a factor of 3–5 compared with a random selection of holdings for on-farm animal welfare inspections. Since there are also false negative classifications, the results of this study should be considered as a priority list for planning and conducting on-farm inspections rather than a conclusive list of holdings with animal welfare issues.

### 4.1 Feature analysis

The importance of farms participating in ethological and/or ecological programmes suggests that these programmes tend to achieve their goals. However, they are a potential source of bias if farms participate in these programmes but do not fully comply with the corresponding production regulations. Geographical location also had an impact on the classification. There might be locally more farms at risk for welfare violations for different reasons, such as production types, local emphasis on the controls, or even traditions. This hypothesis would imply that the local bias of the controllers is not compensated by the classifier. However, our current knowledge does not suggest this explanation to be true and, on the contrary, reinforces the conclusion that there are indeed more holdings at risk in certain regions. Even though the diversity of animal species held was important for pigs, there is a correlation, at least for pigs, between welfare and the number of the animal species reared on one particular livestock unit. For pigs, computed transport duration features were more relevant than the notification discipline.

In both cases, some of the features barely have an impact. This might be because they are not good proxy information and, therefore, are not relevant because the feature is badly designed and does not carry the relevant information. A feature that would be too noisy would also be classified as uninformative, even if it was a priori relevant. Distinguishing the cause of the unimportance is not trivial. The fact that the number of features can be lowered to 75% of the decision without changing the major outcomes, and the previous statement seem to suggest that there is a scope for improvement in the choice and design of the features. The use of further databases more closely involved with health and welfare would also be beneficial. However, these databases were either not yet ready for implementation or data quality was insufficient when the study was conducted.

Initially, it was expected that dynamics in the animal population over time would play a role. For both animal species, none of these features rank among the top 50 most important ones. The clustering of the population dynamics may be too complex and noisy to recover meaningful information or is already encoded in other parameters, such as the type of holding.

A small number of holdings is consistently misclassified. These holdings mostly did not participate in ethological programmes, i.e., some of the holdings not subscribing to BTS/RAUS were classified as at risk consistently across the different training runs, even if they were not at risk. This effect is especially strong for holdings with dairy cows that do not take part in programmes regarding ecological services. This bias might be due to the small size of the training set (and thus more data could compensate the effect), but it could also be due to excessively high sensitivity of the participation in programmes. This could be a manifestation of the limitation of proxy data and should be investigated in future work. Local or traditional habits, or convictions of the farmers, given animal welfare violations, have a large influence on the results. Livestock farmers who participated in extended ecological service programmes may also be more sensitive to the health and welfare of their herd, or they invest more time for observation and care, and this is not reflected in the proxy information at disposal.

### 4.2 Classification analysis

Cattle farms were classified just as accurate as cattle farms. Due to the lower data coverage compared with cattle, this was somewhat unexpected. However, the finding might be the result of the smaller sample size; pig farms are quite homogeneous compared with the much more diverse cattle production systems.

When feature importance is combined with the individual prediction, the relevant features can be computed for each holding. The features can be ranked according to their contribution to the final decision of individual holdings and explained the classification for these individual holdings.

For both species, the feature space is dimensionally large. Giving a one-dimensional range of value for an arbitrary feature that implies a classification at risk is excessively difficult. It is not meaningful because of the marginalisation over the other features. For example, a lower mortality rate is always better; however, some circumstances could explain certain levels that could be considered normal. The same mortality rate at a holding with young calves or heifers cannot be expected. The inspection tools allow to consider these effects if they were picked up by the classifier during the training phase. The analysis of the classification(s) should not lead to naïve or oversimplified conclusions. There might be strong correlations, but they do not imply causation. A prediction “at risk” does not imply that there is indeed a problem, but merely the probability of welfare violations is higher. Inspecting feature also provides possible areas of improvements for holdings, which can have a positive impact on animal health and welfare, based on proxy data. Such schemes would be difficult to manipulate or falsify. Forcing few features might not have the desired impact. Most of the features are based on the notifications, which are hard to falsify. Other features are redundant and thus protected against manipulation.

## 5 Conclusion

The outcomes of the current study demonstrate that by combining historical inspection data with other existing livestock databases and applying machine learning algorithms, it is possible to identify holdings with a higher risk of welfare violations and recommend these farms for an on-farm animal welfare inspection. The models achieved sensitivities in excess of 80% and a precision of 12–18%. This means that the index correctly discovers most of the holdings, which have a history of health and welfare violations. Most important feature groups for both of the studied species were the participation in ethological and ecological programmes and AMD features such as departure notifications, notification history and delay, and, for pigs, the computed transport duration. These findings suggest that supposedly trivial aspects such as notification discipline or the naming of animals have positive effects on complex processes such as health and welfare. Future work should focus on integrating further data sources and improving feature design to further improve sensitivity and precision of the models. As various sub-control points of the on-farm inspection protocol (e.g., lameness and body condition) were not labelled individually, the risk index cannot be applied to indicate the likelihood for violations of specific control points but only combined non-specifically across all of them. Nevertheless, the current models can already be used to create priority lists that can be utilised for planning and conducting risk-based inspections of cattle and pig farms to determine the true animal welfare status.

## Data availability statement

The data analysed in this study is subject to the following licences/restrictions: the raw data supporting the conclusions of this article is confidential and will be made available in anonymized form by the authors upon request. Access to public data is regulated by public law. Requests to access these datasets should be directed to the Federal Food Safety and Veterinary Office (FSVO) as well as the Federal Office for Agriculture (FOAG).

## Author contributions

BT: Conceptualization, Data curation, Investigation, Methodology, Writing – original draft, Writing – review & editing, Project administration. TK: Writing – original draft, Writing – review & editing, Data curation, Formal analysis, Investigation, Methodology, Visualization. GS-R: Writing – original draft, Writing – review & editing, Conceptualization, Funding acquisition, Project administration, Supervision, Resources. SR: Conceptualization, Funding acquisition, Methodology, Project administration, Supervision, Validation, Writing – original draft, Writing – review & editing, Resources.
